# Rheumatic fever and long-term use of benzathine penicillin as possible risk factors for extensive macular atrophy with pseudodrusen in a Brazilian cohort

**DOI:** 10.1186/s40942-024-00592-y

**Published:** 2024-10-11

**Authors:** Carlos Augusto Moreira-Neto, Rafaella Atherino Schmidt Andujar, John Chii Tyng Chao, Huber Vasconcelos, Fábio Eduardo Eberhardt Alves, Gabriela Doná Rodrigues, Bruno Hirt, Jayme Arana, Eduardo Cunha Souza, André Maia, Juliana Maria Ferraz Sallum, Carlos Augusto Moreira

**Affiliations:** 1 Hospital De Olhos Do Paraná, 483, Presidente Taunay St. Alameda Presidente Taunay, 483 Batel, Curitiba, CEP 80420-180 PR Brazil; 2 Retina Clinic, Rua Estados Unidos, São Paulo, 1881, CEP 01427-001 SP Brazil; 3 University of São Paulo, Rua da Reitoria, 374 Butantã, São Paulo, CEP: 05508-220 SP Brazil; 4 Federal University of São Paulo, Rua Botucatu, 720. Vila Clementino, São Paulo, CEP 04023-062 SP Brazil; 5 Ocular Genetics Institute, Rua Helena, 335 Conjunto 92, Vila Olímpia, São Paulo, CEP 04552-050 SP Brazil

**Keywords:** Extensive macular atrophy with pseudodrusen-like appearance, EMAP, Rheumatic fever, Benzathine penicillin, Macular atrophy, Electroretinography

## Abstract

**Background:**

Although there has been a large increase in the number of extensive macular atrophy with pseudodrusen (EMAP) cases, the basic aspects of this disease remain unknown. Brazilian patients have a common past history of rheumatic fever (RF) and/or benzathine penicillin (BP) treatment possibly related to the disease. We analyzed how RF and BP might be correlated with EMAP in Brazilian patients.

**Design:**

Observational, retrospective, case-control study.

**Methods:**

The databases of three private eye clinics in Brazil were searched for patients with an EMAP-like appearance. Each patient was asked about a previous history of RF and/or long-term use of BP. Patients underwent best-corrected visual acuity (BCVA) measurement, color fundus imaging, fundus autofluorescence (FAF) imaging, optical coherence tomography (OCT) imaging, and electroretinography (ERG). The following characteristics were analyzed: subretinal drusenoid deposits (SDD), pigment mottling, retinal pigment epithelial/basement membrane (RPE/BM) separation, outer retinal or RPE atrophy, and identification of a paving stone-like appearance. The choroidal thickness was measured using enhanced depth imaging OCT. The central atrophic area was measured manually on ultra-wide-field FAF.

**Results:**

A total of 154 eyes of 77 patients (women, 66.2%; mean age, 58.6 years) with EMAP were included; 90.9% of patients were diagnosed with RF; 94.8% had been treated with BP and treatment was started at an average age of 7.3 years (mean duration, 11.8 years). The treatment duration was significant for the area of atrophy (*P* = 0.027) in which each 1-year increase in treatment duration led to an average reduction of 6.91 mm^2^ in area. The age at diagnosis of RF was significant (*P* = 0.026) for SDD. The increase of 1 year in the diagnosis of RF (late disease) led to a reduction of 24% in the chance of central SDD being present. On OCT, 65.5% eyes had SDD and more than 70% had a split RPE/BM and outer retinal or RPE atrophy. The choroidal thickness in patients with EMAP was significantly (*P* < 0.001) thinner than the control group. The ERG was abnormal in all eyes.

**Conclusion:**

These findings may suggest a relation between RF and EMAP in Brazilian patients. Patients with EMAP should be questioned about a history of RF.

## Introduction

Hamel et al. in 2009 first described extensive macular atrophy with pseudodrusen (EMAP) as an uncharacterized macular disorder that included macular atrophy, drusen-like deposits in the mid-periphery, and paving stones in the far periphery [1].

EMAP is a rare clinical entity that represents a form of macular atrophy distinguished by its rapid progression and severe impact on vision, typically affecting individuals around the age of 55 years [1, 2]. This condition results in legal blindness within a relatively short span of 3 to 10 years [1, 2]. The symptoms of EMAP typically start with progressive night blindness, swiftly progressing to bilateral and central visual deterioration [2].

EMAP manifests as chorioretinal atrophy that spreads to the temporal arcades and notably involves the fovea; it is accompanied by widespread pseudodrusen across the posterior pole and peripheral retina [1, 2]. The macular atrophy is typically predominant in the vertical axis and occurs simultaneously and symmetrically in both eyes. EMAP lesions are consistently surrounded by extensive pseudodrusen with occasional paving stone lesions in the far peripheral retina [2].

Although EMAP resembles the dry form of age-related macular degeneration (AMD), it exhibits distinct clinical features such as natural progression and the presence of subretinal deposits or paving stones in the far periphery [3–8]. Unlike atrophic AMD, EMAP manifests earlier, affects both eyes simultaneously, and the central vision rapidly deteriorates within a few years [2].

To date, EMAP pathophysiology and risk factors remain unknown. Some authors believe EMAP could be a distinct clinical entity caused by a specific pathogenic mechanism [2]. In 2016, Douillard et al. reported that EMAP is associated with marked complement pathway dysfunction and inflammation.

Rheumatic fever (RF) is an inflammatory autoimmune disease associated with group A β-hemolytic streptococcal infection, characterized by inflammation with multisystem involvement [9, 10].

RF most likely results partly from the production of autoreactive antibodies and T-cells that cross-react with components of group A streptococcus and host tissues [11]. As with any autoimmune disease, several monoclonal antibodies are activated systemically. Lerner et al., in 1995, investigated anti-streptococcal monoclonal antibodies (MAbs) for cross-reactivity with the human eye and found a novel cross-reaction of anti-streptococcal MAbs with the retina [12]. The authors suggested that immune responses to specific epitopes of common pathogens, such as group A streptococci, cross-react with S-Ag and structures in the human eye [12].

Primary prevention of RF consists of properly treating streptococcal pharyngitis with benzathine penicillin (BP) and secondary prevention with its long-term administration [9].

The goal of this study was to characterize the demographic, clinical and multimodal imaging, and functional data of patients with EMAP in Brazil. We also analyzed RF and/or long-term use of BP as risk factors for an EMAP-like appearance.

## Methods

### Study design and participants

We conducted an observational retrospective cohort study in which we searched the databases of three private eye clinics in Brazil for patients with an EMAP-like appearance between 2022 and 2024. The institutional Ethics Committee approved the study, which was conducted according to the tenets of the Declaration of Helsinki.

The inclusion criteria included patients who had been diagnosed with an EMAP-like appearance, according to Romano et al. [13] and without applying an age cut-off for symptom onset, according to Antropoli et al. [14]. Patients who presented with other ophthalmologic diseases, such as diffuse trickling geographic atrophy or non-diffuse trickling geographic atrophy that could confuse the EMAP diagnosis were excluded [15]. 

Demographic characteristics such as age, sex, ethnicity, age of onset of visual symptoms, and medical history were collected from the medical records. Each patient was asked about a previous history of RF and/or long-term treatment with BP. Patients also were questioned about previous and current histories of heart disease, nephropathy, or other systemic diseases; smoking habits; family medical history; and daily prescription drugs. Patients also were questioned about a previous diagnosis of RF; the age of diagnosis; the main reason to confirm the diagnosis; systemic involvement (cardiac, kidney, central nervous system); medications with which they were treated; and the ages at which treatment started and ended. The patients with missing data in their medical history were contacted by phone.

Regarding ophthalmologic information, patients were asked about the main symptoms, especially loss of peripheral vision and nyctalopia.

The best-corrected visual acuity (BCVA) (logarithm of the minimum angle of resolution [logMAR]), color fundus images, fundus autofluorescence (FAF), optical coherence tomography (OCT), and electroretinography (ERG) were collected from medical charts. Both eyes were considered for the descriptive analysis.

It is worth noticing that not all patients had data on each characteristic analyzed in the study. Therefore, each imaging test was analyzed and presented independently.

### Imaging analysis, measurements, and outcome variables

**Fig. 1:**
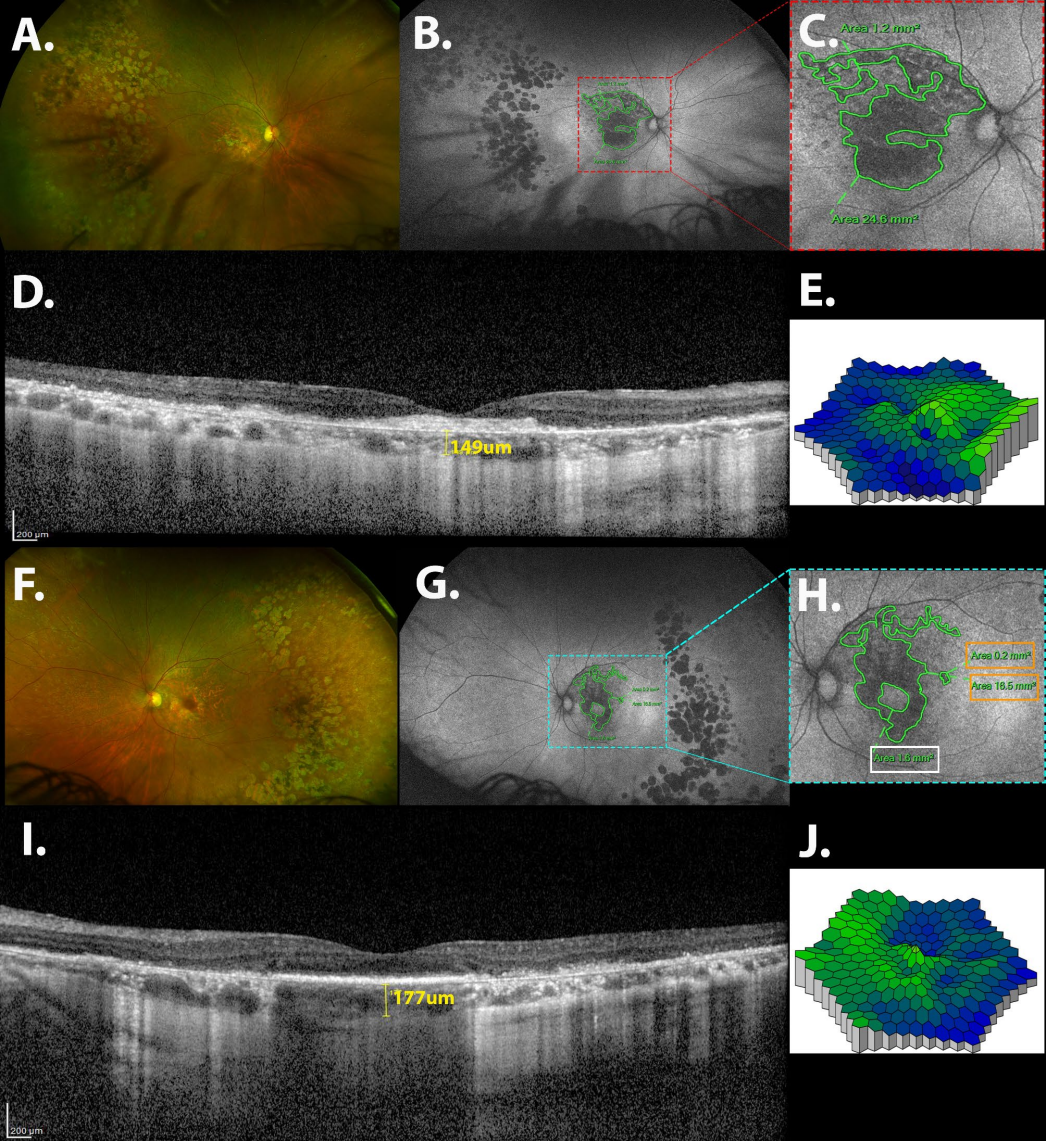
Multimodal imaging analysis of the anatomic and functional findings in a Brazilian patient with an EMAP-like appearance. A 60-year-old woman had a BCVA of < 1.3 in the right eye with stage 3 EMAP; the BCVA in the left eye with stage 2 EMAP was 1.0 logMAR. (**A**, F) An UWF color fundus photograph shows central retinal atrophy, peripheral subretinal deposits, and a paving stone-like appearance in the right and left eyes, respectively. (**B**,** G**) UWF-FAF images show central and peripheral retinal atrophy. (**C**,** H**) Magnified images of the central retinal atrophy on FAF are seen in (**B** and *G*). (**C**) Two major areas of atrophy are seen. The sum of the central atrophic areas is 25.8 mm^2^. (**H**) In the left eye, the atrophy spares the foveal center. Therefore, two atrophic areas (orange rectangles) were added, and the total was subtracted from the area that spares the central region (white rectangle). As a result, the total central atrophic area in the left eye is 15.1 mm^2^. (**D**, **I**) The EDI-OCT images of the left and right eyes, respectively. (**D**) The image shows loss of the outer retina associated with RPE atrophy and a central area with subretinal fibrosis. The choroidal thickness is 149 μm. (**I**) The image shows loss of the outer retina. The RPE is present in the central area. RPE atrophy is seen in the paracentral area. The choroidal thickness is a 177 μm. (**E**,** J**) The mfERG shows a decreased P1-wave amplitude in the right and left eyes, respectively

Color fundus imaging was performed to analyze the retinal findings using either ultra-wide field (UWF) images (Optos California ICG, Marlborough, MA, USA) (Fig. 1A, F) or conventional color fundus photographs (Canon CX-1-USA, Melville, NY, USA). The following characteristics were analyzed: subretinal drusenoid deposits (SDD), posterior pole pigment mottling, and peripheral deposits. Conventional and/or UWF-FAF were useful to define the presence of posterior pole atrophy and a paving stone-like appearance. Experienced retinal specialists (CAMN, HV, JCC) manually measured the central atrophic area (Fig. 1C, H) and vertical and horizontal diameters of patients who had undergone UWF-FAF (Fig. 1B, G) using the Optos “Free Hand” Area Measurement tool. Because the Canon camera does not have an automatic area measurement tool, only the vertical and horizontal diameters were measured. UWF imaging also was helpful to analyze the presence of peripheral subretinal deposits and a paving stone-like appearance.

Further macular information, i.e., SDD, split RPE/BM, outer retina atrophy, RPE atrophy, and measurement of the choroidal thickness, were obtained using a central raster pattern spectral-domain OCT and enhanced depth imaging (EDI)-OCT (Fig. 1D, I) (Spectralis, Heidelberg Engineering, Heidelberg, Germany).

### Electrophysiologic tests

All electrophysiologic tests were recorded using a Roland electrophysiologic system (RETIscan, Roland Consult, Brandenburg an der Havel, Germany) with ERGjet™ lens electrodes (Fabrinal, La Chaux-de-Fonds, Switzerland), which complied with the standards of the International Society for Clinical Electrophysiology of Vision [16]. Multifocal ERGs (mfERG) (Fig. 1E, J) were recorded with a protocol of 61 hexagons in five segments [17]. The stimulus array consisted of 61 hexagonal elements displayed randomly on a cathode-ray tube monitor (frame rate, 60 Hz) at 28 cm, covering a 30-degree field of view visual angle.

### Statistical analysis

In statistical methodology, initially the data were analyzed descriptively.

Comparisons of gender distribution and mean ages between the study and control groups were performed using the chi-square test and Student’s t-test, respectively.

The effects of the demographic and clinical characteristics on dependent variables of a dichotomous nature (measured in the eyes) were assessed via logistic regression of the class of generalized estimating equation models (GEE) [18] to accommodate for a possible dependence between information from the right and left eyes of the same patient. There was no convergence in the estimation of logistic regression models with the regression model, partly because the presence or absence of a dependent variable based on eye laterality was the same in most patients.

For dependent variables of a numerical nature, linear models with regression (or mixed) models were used [19]. The linear regression model with random effects incorporates the effect of each patient in the form of a random model and accommodates a possible dependence between observations of the same patient. This model assumes normality in the data distribution. However, Gelman and Hill [20] pointed out that the departure from normality does not lead to bias in the estimates. For VA, the Tobit model with regression was used [21]. The mixed Tobit regression model is usually used for censored data, which occurs in the data due to the impossibility of quantifying below or above a certain value (in VA, the data correspond to cases < 1.30).

Univariate and multivariate models were adjusted for all outcomes. Due to the limited sample size, predictor variables significant at 10% in the univariate models were considered in the initial multivariate model. Non-significant variables then were eliminated one by one in order of significance (backward method).

Due to the small number of eyes with information on the ERG-multifocal variables, these variables were analyzed descriptively, assuming that the observations of the right and left eyes are independent. Thus, distribution comparisons were performed using the chi-square test or Fisher’s exact test.

A comparison of means was performed using the Student’s t-test or non-parametric Mann-Whitney test in the event of violation of the assumption of normality in the data distribution. Normality in the data distribution was verified using the Kolmogorov-Smirnov test.

For all statistical tests, a significance level of 5% was adopted. Statistical analyzes was performed using SPSS 20.0 (IBM Corp., Version 20.0. Armonk, New York, USA) and STATA 17 (StataCorp. 2021., College Station, Texas, USA) .

## Results

### Characterization of patients in the study group

A total of 154 eyes of 77 patients (66.2% women; average age, 58.6 years; standard deviation [SD], 4.6 years) had an EMAP-like appearance with both eyes affected. All were Caucasian and 22.0% were smokers. We also found that 90.9% of patients had been diagnosed with RF (average age at diagnosis, 7.5 years; SD, 3.4 years). According to the patients, their RF diagnosis in childhood was due to joint pain (78.4%), tonsillitis (35.1%), fever (21.6%), and heart disease (5.4%). Further, 94.8% from 77 patients with EMAP-like appearance had been treated with BP (average age at start of treatment and treatment duration, respectively, 7.3 years; SD, 3.1 years and 11.8 years; SD, 6.7 years). In addition, 23.0% had current heart disease and 56.3% had nyctalopia complaints. Table 1 shows the demographic and clinical characteristics of the EMAP cases.

**Table 1:** Demographic and clinical characteristics of patients

Female, *n* (%)	51/77 (66,2)
Age (years)	
Mean ± SD	58,6 ± 4,6
N	77
**Caucasian**,** n (%)**	77/77 (100,0)
**Diagnosis of RF**,** n (%)**	70/77 (90,9)
**Age of RF diagnosis (years)**	
Mean ± SD	7,5 ± 3,4
N (patients)	45
**BP**,** n (%)**	73/77 (94,8)
**Treatment initiation (years)**	
Mean ± SD	7,3 ± 3,1
N (patients)	67
**Treatment duration (years)**	
Mean ± SD	11,8 ± 6,7
N (patients)	52

**Fig. 2:**
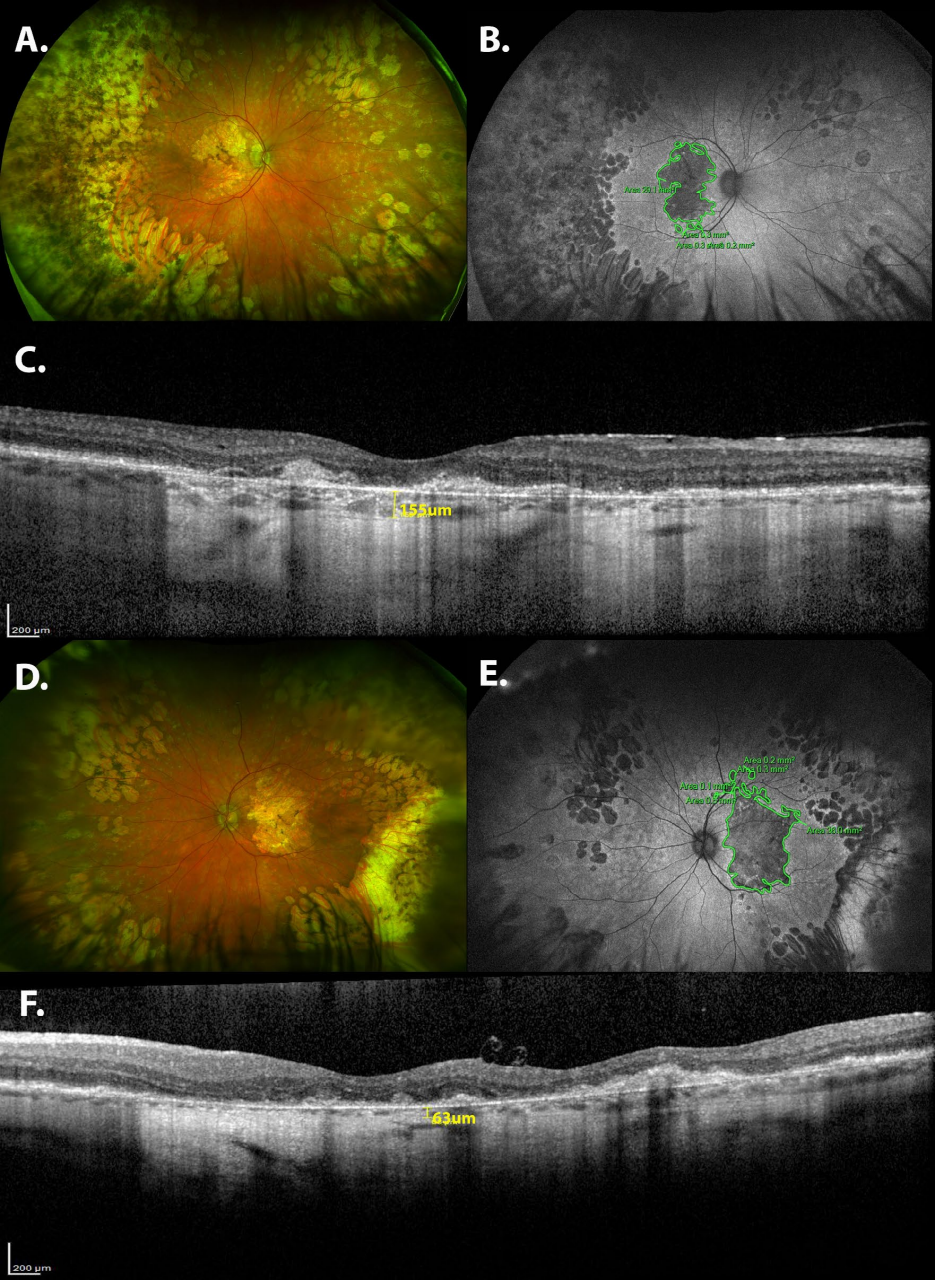
Multimodal imaging analysis of the anatomic findings in a 57-year-old Brazilian man with stage 3 EMAP. The bilateral BCVA is < 1.3 logMAR. (**A**,** F**) An UWF color fundus photograph shows central retinal atrophy, peripheral subretinal deposits, and a paving stone-like appearance in the right and left eyes, respectively. (**B**,** E**) UWF-FAF images show central and peripheral retinal atrophy in both eyes. (B) One major and three small areas of atrophy are seen. The sum of the central atrophic areas is 29.9 mm^2^. (E) One major and four small areas of atrophy are seen. The sum of the central atrophic areas is 37.1 mm^2^. (**C**,** F**) EDI-OCT images of the right and left eyes, respectively, show loss of the outer retina associated with RPE atrophy. (**C**) A paracentral area with subretinal fibrosis is identified. The choroidal thickness is 155 μm in (**C**) and 63 μm in the left eye

The mean BCVA in all 154 affected eyes were 0.7 (SD ± 0.5) logMAR. Macular depigmentation and SDD were seen in 87.1% and 71.4% of the eyes, respectively (Table 2). In addition, 75.7% and 27.1%, respectively, had central atrophy and a paving stone-like appearance in the periphery. OCT images showed that 65.5% had SDD and 72.9% had split RPE/BM, outer retinal atrophy, or RPE atrophy. Figure 2 shows a case of late-stage EMAP.

**Table 2:** Ophtalmological clinical and imaging chacacteristics

**LogMar BCVA**	
Mean ± SD	0,7 ± 0,5
N (eyes)	154
**Conventional or UWF retinography**,** n (eyes-%)**	
SDD	100/140 (71,4)
Peripheral deposits	66/140 (47,1)
Macular depigmentation	122/140 (87,1)
**Convencional or UWF autofluor**	
**Central atrophy**,** n (eyes-%)**	106/140 (75,7)
**Area (mm2)**	
Mean ± SD	33,2 ± 25,0
N (eyes)	32
**Vertical Size (mm)**	
Mean ± SD	6,7 ± 2,6
N (eyes)	76
**Horizontal Size (mm)**	
Mean ± SD	5,1 ± 2,5
N (eyes)	76
**Relation vertical size/ horizontal size**	
Mean ± SD	1,4 ± 0,3
N (eyes)	76
**OCT**	
**SDD**,** n (%)**	80/122 (65,5)
**Split RPE/BM**,** n (%)**	89/122 (72,9)
**Outer retinal atrophy**,** n (%)**	94/122 (77,0)
**RPE atrophy**,** n (%)**	85/122 (69,6)
**Central choroidal thickness**	
Mean ± SD	130,3 ± 60,8

### Association between ophthalmologic characteristics and variables related to RF

The ophthalmologic characteristics and variables related to RF analyzed were sex, age, diagnosis of RF, age at diagnosis of RF, past treatment with BP, age at start of treatment, and treatment duration. These results are presented in the following subsection: Conventional or UWF retinography: central SDD, UWF autofluorescence: area, Conventional or UWF autofluorescence: vertical size, Functional analysis: mfERG.

### Conventional or UWF retinography: central SDD

This analysis used a logistic regression model to study the presence of central subretinal drusenoid deposits, the only variable that proved statistically significant was the age at the diagnosis of rheumatic fever (*P* = 0.026).

The increase of 1 year in the diagnosis of RF (late disease) led to a reduction of 24% in the chance of the presence of central SDD.

### UWF autofluorescence: area

**Table 3:** Results of linear regression model with random effects for area (mm^2^) in patients with the diagnosis of RF

	Univariate Model				Initial Multivariate Model	
	Gross Profit Coefficient (95% CI)	**P**	No. Eyes		Adjusted Coefficient (95% CI)	**P**
Treatment duration (years)^1^	-1.51 (-3.22-0.21)	0.086	32		-6.91 (-13.06 a -0.77)	0.027

P = descriptive level of the linear regression model with random effects

^1^Indicates patients treated with BP

In the multivariate model for the area, with age at the diagnosis of RF and the time of treatment as predictors, time proved to be significant. Thus, only the treatment duration was significant (*P* = 0.027) adjusted by the age at RF diagnosis; thus, the 1-year increase in treatment duration led to an average reduction of 6.91 mm^2^ (Table 3).

### Conventional or UWF autofluorescence: vertical size

Based on the linear regression model with random effects for vertical size, only the treatment duration was significant (*P* = 0.049). Thus, a 1-year increase in the treatment duration led to an average reduction of 0.17 mm in vertical axis.

### Functional analysis: mfERG

All 19 patients who underwent electrophysiology tests had abnormal mfERG results. mfERG showed a decreased P1-wave amplitude. When focusing on ring 2 of the mfERG, it is important to mention that the mean age at diagnosis of RF (*P* = 0.029) and the mean age at the start of treatment (*P* = 0.032) in the group without changes were higher than the group with changes.

### Non-statistically significant predictors

The analysis showed that the presence of a paving stone-like appearance, macular depigmentation, horizontal size and vertical size/horizontal size ratio were not significant predictors of an EMAP-like appearance.

## Discussion

The clinical characteristics of Extensive Macular Atrophy with Pseudodrusen (EMAP) are marked by extensive, sometimes symmetric, macular atrophy – multilobular borders and a faint/grayish hypoautofluorescent aspect- with surrounding pseudodrusen and paving stone lesions. These lesions can appear sporadically in the far peripheral retina, and the atrophy is more pronounced along the vertical axis than the horizontal. EMAP is distinct from age-related macular degeneration (AMD), as it lacks the significant horizontal atrophy and the hard and soft drusen typical of AMD. EMAP progression differs from atrophic AMD, which starts with small, round atrophic spots that gradually merge, often sparing the fovea for an extended time [3–8]. 

EMAP affects middle-age patients younger than 60 years of age. These patients usually have severe vision loss, which also was evident in the current study. The disease is characterized by rapid progression. Thus, due to these characteristics and its potential socioeconomic impact, EMAP has caught the attention of the scientific community. However, the etiology of EMAP remains unknown. In 2023, Watanabe et al., who reported the retinal electrophysiologic findings in patients with EMAP [22], also mentioned that their 18 patients had a previous history of RF. Despite that, to the best of our knowledge, no in-depth studies have clarified the possible relation between an EMAP-like appearance and RF and/or long-term use of BP. The current results showed that 90.9% of the patients had a previous history of RF and 94.8% had been treated with BP for an average of 11.8 years.

Cunningham et al. and Lerner et al. studied the autoimmune implications of group A β-hemolytic streptococcus, specifically focusing on rheumatic fever (RF). They identified that microbial antigens, like the M proteins of Streptococcus pyogenes, share epitopes with human tissues, potentially leading to autoimmune reactions. The M protein, a key virulence factor, triggers strong immune responses and may act as a superantigen. These studies highlighted that antibodies against streptococcal M proteins cross-react with human retinal tissues, suggesting that autoimmune responses against these pathogens could also target structures in the human eye [9, 11, 12]. 

In 1995, Lerner et al. studied the immunologic cross-reactivity between the uveitogenic retinal S-Ag and streptococcal M protein, the major virulence factor of group A streptococci [12]. The streptococcal M protein, which shares epitopes with proteins in host tissues such as the heart, joints, and skin, may be responsible for the development of post-streptococcal sequelae following infection [12].

All these studies are helpful to explain why more than 90% of our patients with an EMAP-like appearance had RF and how this disease could be a risk factor for EMAP in Brazil.

Almost every patient with RF had undergone long-term treatment with mainly BP [23, 24]. Among our patients, 94.8% mentioned long-term treatment with BP. In 2021, Lin et al. conducted a systematic review and suggested a link between the intestinal microbiota and AMD pathogenesis [25]. Based on that, we analyzed a possible relationship between long-term treatment with BP and an EMAP-like appearance in patients with RF. UWF-FAF analysis showed that treatment duration was associated significantly with the area of atrophy. Surprisingly, a 1-year increase in treatment duration led to an average reduction of 6.91 mm^2^ in the area. The same was found for the vertical axis. A 1-year increase in the treatment duration led to an average reduction of 0.17 mm in the vertical axis. These findings suggested that longer use of BP could result in a smaller area of atrophy in eyes with an EMAP-like appearance in patients with RF in Brazil. The different results found by our group compared to those of Lin et al. could be explained by the fact that they included patients with AMD, and we included only patients with EMAP-like appearance.

In the current study, the vertical axis (mean, 6.8 mm) was longer than the horizontal axis (mean, 5.3 mm). These findings have was already been published in the literature and are a well known characteristic of EMAP [2, 13]. We also found that patients with an EMAP-like appearance had significantly thinner choroidal thickness, as described by Antropoli [14]. 

Recently, Romano et al. reported the initial changes preceding the onset of macular atrophy and introduced the inaugural disease classification [13]. In summary, stage 1 is typified by widespread pseudodrusen-like lesions with negligible or minimal RPE atrophy. With disease advancement, the macular atrophy extends significantly (stage 2) and ultimately encompasses the fovea (stage 3) [13]. Regarding clinical characteristics, in the current study, we detected that the age at diagnosis of RF was significant for SDD development, using retinography. The increase of 1 year in the diagnosis of RF (late diagnosis) led to a reduction of 24% in the chances of the presence of central SDD.

Our central raster OCT scans showed that 65.5% had SDD and 72.9% had a split RPE/BM, outer retinal atrophy, or RPE atrophy. The smaller number of eyes with SDD on OCT compared to retinography could be explained because SDD spread extensively beyond the arcades, an area that is not scanned by central raster OCT. Subretinal fibrosis also was seen in a few eyes. Romano et al. described that subretinal fibrosis was seen in late-stage EMAP. According to those authors, foveal involvement takes place later in the course of the disease and frequently manifests as subretinal fibrosis with significant visual loss [13].

In the current study, all patients with an EMAP-like appearance who underwent **mfERG** test showed abnormal results. These findings were the same as those reported by Watanabe et al. [22]. The decreased P1-wave amplitude reinforced that the outer retina function is damaged in patients with an EMAP-like appearance. Considering ring 2 of the mfERG, it is important to mention that the mean age at diagnosis of RF and the mean age of treatment initiation (*P* = 0.032) in the group without alterations were higher than the group with changes. This functional finding endorsed the significant structural findings described previously.

The current study was limited by its retrospective design, which resulted in not every patient having all multimodal images available for analysis. Also, in our study group, 100% of patients were Caucasian, which does not represent the Brazilian population. However, it is important to highlight the majority of these patients live in the south of Brazil, where most of the population is Caucasian. We may also point that missing previous medical history was collected by phone. The fact that patients are, on average, 58 years old and that rheumatic fever occurred or started in childhood may be a limitation due to the difficulty in accurately recalling information.

Despite those limitations, our findings suggested a possible relation between RF and EMAP in Brazilian patients. Equally important, a significant relation was seen between treatment duration with BP and a smaller atrophic area. This hypothesis is strengthened by the functional finding on ring 2 of the mfERG. The immunologic studies of Cunningham et al. and Lerner et al. support this possible association [11, 12]. However, large prospective studies with genetic analysis and better data collection conditions are needed to support this theory and to clarify if RF is a risk factor for EMAP or if EMAP is just a late stage of the spectrum of RF. The current findings should also alert countries with high incidence rates of RF and encourage retinal specialists to question their patients with EMAP about a previous history of RF during childhood.

## Data Availability

No datasets were generated or analysed during the current study.

## Abbreviations

EMAP: extensive macular atrophy with pseudodrusen-like
RF: rheumatic fever
BP: benzathine penicillin
BCVA: best-corrected visual acuity
FAF: fundus autofluorescence
SDD: subretinal drusenoid deposits
RPE: retinal pigment epithelium
BM: Bruch’s membrane
UWF: ultra-wide field
ERG: electroretinogram
mfERG: multi-focal electroretinogram
AMD: age-related macular degeneration
GEE: estimating equation models
VA: visual acuity
SD: standard deviation
DTGA: diffuse trickling geographic atrophy
OR: odds radio
CI: confidence interval
